# Exploring taxonomic diversity and biogeography of the family Nemacheilinae (Cypriniformes)

**DOI:** 10.1002/ece3.5553

**Published:** 2019-08-28

**Authors:** Weitao Chen, Jiping Yang, Yuefei Li, Xinhui Li

**Affiliations:** ^1^ Pearl River Fisheries Research Institute Chinese Academy of Fishery Sciences Guangzhou China

**Keywords:** COI, mitochondrial genomes, Nemacheilidae, phylogeny, recombinase‐activating gene proteins 1, taxonomic problem

## Abstract

Nemacheilidae, in the superfamily Cobitoidea, is comprised of many of morphologically similar fish species that occur in Eurasian water bodies. This large group shows inconsistencies between traditional morphological taxonomy and molecular phylogenetic data. We used mitochondrial genomes, recombinase‐activating gene proteins 1 (RAG1) and the mitochondrial cytochrome *c* oxidase I gene (COI) to study the phylogenetic relationships among Nemacheilidae species using Bayesian inference and maximum likelihood approaches. Phylogenetic analyses based on mitogenomes provided support for two clades (I and II). The mitogenomes, RAG1, and COI results indicated that several species and genera were not consistent with the traditional morphological subdivisions. The two clades inferred from mitogenomes showed clear geographical patterns. The Tibetan Plateau, Hengduan Mountains, and the Iran Plateau may act as a barrier dividing the clades. The estimated timing of clades separation (36.05 million years ago) coincides with the first uplift of the Tibetan Plateau. We conclude that the geological history of the Tibetan Plateau played a role in the diversification and distribution of the Nemacheilidae taxa. These results provided a phylogenetic framework for future studies of this complex group.

## Graphical abstract

In this study, we combined two novel mitochondrial (mtDNA) genomes and 56 published mtDNA genomes of Nemacheilidae species to reconstruct the robust phylogenetic trees for Nemacheilidae species and discuss taxonomic issues and biogeographic history occurred within this family. The results demonstrate many species and even genus are not congruent with the traditional morphological subdivision and find that the geological history of the Tibetan Plateau plays a critical role in driving the diversification and distribution of the Nemacheilidae taxa.

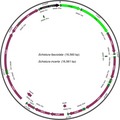

## 1 INTRODUCTION

The Nemacheilidae is a large family with more than 600 known fish species in 48 genera (Eschmeyer & Fong, 2015). Several new Nemacheilidae species have been continuously reported in the past decade (Du, Chen, & Yang, 2008; Erk‐Akan, Nalbant, & Zeren, 2012; Hu & Zhang, 2010; Kosygin, 2012; Zheng, Du, Chen, & Yang, 2009). More new species are expected to be named in this family. Nemacheilids are small benthic fish mainly distributed in Eurasian waters (http://www.fishbase.com), with the exception of the genus *Afronemacheilus* that occurs in Africa. Most Nemacheilid species are found in Asia (Kottelat, 2012; Zhu, 1989), and this suggests that Asia is the distribution center of this group (Eschmeyer & Fong, 2015).

There are many morphologically similar species within Nemacheilidae (Nalbant & Bianco, 1998). The species with significant morphological variation and widespread distributions present taxonomic problems. Morphological variation is often inconsistent with the proposed molecular phylogenetic relationships (Liu et al., 2012; Prokofiev, 2009; Sgouros, 2016; Tang, Liu, Mayden, & Xiong, 2006; Zhu, 1989). For example, *Schistura* species do not display monophyly but instead show polyphyly (Liu et al., 2012; Nalbant & Bianco, 1998; Sgouros, 2016; Tang et al., 2006). Several *Homatula* species exhibit either paraphyly or polyphyly (Min, Chen, Yang, Winterbottom, & Mayden, 2012). Studies including morphological traits and phylogenetic inference are needed to unravel the complex species relationships among the Nemacheilidae.

Previous studies focused on the phylogeny of a small number of species or specific genera within the Nemacheilidae (Bohlen & Lechtova, 2009; Jamshidi, Abdoli, Sherafatian, & Golzarianpour, 2013; Liu et al., 2012; Liu, Zhang, Tang, & Liu, 2010; Min et al., 2012; Sgouros, 2016; Tang et al., 2006; Wang et al., 2016). Tang et al. (2006) studied the phylogeny of the Cobitoidea based on the mitochondrial cytochrome *b* gene and control region, but included only 20 Nemacheilidae species. Wang et al. (2016) studied the phylogenetics of *Triplophysa* using the entire mitochondrial genome. Since most studies have involved limited species, genera, sample sizes, or the number of molecular characters, many phylogenetic patterns could be missed. Additionally, phylogenetic analyses using more species, genus, larger sample sizes, and larger molecular datasets are needed to determine the phylogenetic relationships among Nemacheilidae species.

Understanding the factors shaping the diversity of taxa is important in evolutionary biology. Nemacheilidae species have limited dispersal ability and are unable to readily cross mountains and rivers. As such, the species may provide clues of earlier geological movements. Wang et al. (2016) argued that the Early Miocene uplifts of the Tibetan Plateau led to divergence of the genus *Triplophysa*. After the collision of the Indian plate with Asian plate in the early Cenozoic, the Tibetan plateau began its uplift (Chung et al., 1998; Ruddiman, 1998; Tapponnier et al., 2001; Yin & Harrison, 2000). The uplift of the plateau changed the landscape of the Asian continent and was the key driving force behind long‐term Cenozoic climate change (An, Kutzbach, Prell, & Porter, 2001; Manabe & Broccoli, 1990). Nemacheilids likely experienced the effects of this geologic event because their current distributions are related to geological features such as the Tibetan Plateau, Tarim basin, and the Himalaya Mountains. An accurate estimation of phylogeny and divergence times of living Nemacheilids provides evidence for the influences of orogenic movements.

In this study, we sequenced and annotated two complete mitochondrial genomes of *Schistura fasciolata* and *Schistura incerta* using Illumina MiSeq. We combined our data with 56 complete mitochondrial genomes of Nemacheilidae species in GenBank and reconstruct the robust phylogenetic trees for Nemacheilidae species. We used these data to discuss taxonomic issues within the Nemacheilidae. The recombinase‐activating gene proteins 1 (RAG1) and the mitochondrial cytochrome *c* oxidase I gene (COI) were also used to study the taxonomy of this family. We then discuss how the orogenic movements in Asia appear to have contributed to Nemacheilidae diversity.

## 2 METHODS AND MATERIALS

### 2.1 Specimen collection, DNA extraction, and RAG1 amplification

*Schistura fasciolata* was collected during May in 2015 from the Xinfengjiang River (24.07N and 114.21E), a tributary within the Pearl River drainage. *Schistura incerta* was sampled from an independent river named the Nanliujiang River during July in 2018. A bit of fin tissues of the two species were preserved in 100% ethanol, then stored at −20°C after being transported to the laboratory until used for further DNA extraction.

Total genomic DNA was extracted from fin tissue using a standard phenol/chloroform extraction method. RAG1 fragment for the two species was amplified using the primers RAG1F1 and RAG1.4090R1 (Liu et al., 2012). The amplification of genomic DNA was conducted in a 25 µl reaction with an initial denaturation period of 4 min at 94°C followed by 34 cycles of 94°C for 30 s, primer‐specific annealing temperature of 52°C for 40 s, 72°C for 90 s and a single final extension at 72°C for 7 min. The amplified fragments were purified using 1.2% low‐melting agarose gel electrophoresis and sequenced with the identical primer pair using an ABI PRISM 3700 (Applied Biosystems) automatic DNA sequencer.

### 2.2 Mitochondrial genome amplification, sequencing, assembly, and annotation

Total genomic DNA was extracted from 100 mg fin tissues using a Genomic DNA Isolation Kit (QiaGene) according to the manufacturer's instructions. Complete mitochondrial genome sequencing was performed on the Illumina MiSeq platform (Illumina Inc). The Illumina raw sequence reads were edited using the NGS QC Tool Kit v2.3.3 (Patel & Jain, 2012), with a cutoff value of 50 and 20, respectively, for percentage of read length and PHRED quality score. High‐quality reads were assembled into contigs using the de novo assembler SPAdes 3.9.0 (Bankevich et al., 2012), utilizing a *k*‐mer set of 93, 95, 97, 103, 105, 107, and 115. The de novo contigs were then assembled into complete mitochondrial genomes by further connection using SOAPdenovo (Luo et al., 2012). The harvested mitogenome sequences of *S. fasciolata* (16,560 bp) and *S. incerta* (16,561 bp) were deposited in GenBank (KY404236 and MK361215).

### 2.3 Sequences collection

The mitochondrial genomes of 56 Nemacheilidae species belonging to 16 genera were downloaded from GenBank (Table S1). The distributions and classification system of each species were collected and verified from FISHBASE (http://fishbase.sinica.edu.tw) and literatures (Kottelat, 2012; Zhu, 1989). Two species from the Balitoridae, two species from the Botiidae, and five species from the Cobitinae were included in our analyses. Two species from the Catostomidae derived from GenBank were selected as outgroups (Tang et al., 2006; Table S1).

The RAG1 sequences of 55 Nemacheilidae species belonging to 18 genera were obtained from GenBank (Table S2). The COI sequences of 148 Nemacheilidae species in 26 genera were downloaded from the BOLD system (http://www.boldsystems.org). The COI sequences of *S. fasciolata* and *S. incerta* were extracted from their mitochondrial genomes. The taxonomic information, accession numbers, and distributions of each species are provided in Table S3. Species from the Balitoridae, Botiidae, Cobitinae, and Vaillantellidae were included in RAG1 and COI analyses. Species from the Catostomidae were chosen according to Tang et al. (2006).

### 2.4 Mitochondrial genomes

A total of 68 mitochondrial genomes including the two outgroups were analyzed. We aligned the mitochondrial genomes using MUSCLE (Edgar, 2004) and then extracted 22 tRNA genes, 13 protein‐coding genes (PCGs), and two ribosomal RNA genes manually. We omitted control regions of the mitochondrial genomes from the final matrix, as some species did not possess this gene fragment. We detected a signal of saturation at the third codon positions of the PCGs using Xia's method in DAMBE5 (NumOTU = 32, *Iss* = 0.602, *Iss*.*c* Asym = 0.555, *p* < .0001; Table S4; Xia, 2013; Xia & Lemey, 2009). We ran subsequent analyses without the third codon positions of the 13 PCGs. We partitioned the sequence alignment into five subsets: two for 1st and 2nd codon positions of the 13 PCGs, two for the 12s and 16s rRNA genes, and one for the 22 tRNA genes. The optimal partitioning scheme and the best‐fit nucleotide substitution model for each partition of the mtDNA molecules were estimated using the software PartitionFinder (Lanfear, Calcott, Ho, & Guindon, 2012).

The phylogenetic relationships were reconstructed using Bayesian inference (BI) and maximum likelihood (ML) techniques. The BI analyses were performed using MrBayes 3.1.2 (Ronquist & Huelsenbeck, 2003). Four independent runs were performed for 20 million generations. The phylogenetic trees were sampled every 1,000th generation, which resulted in 20,000 trees, and the first 25% were discarded as burn‐ins. Convergence of the BI analyses was assessed by the average standard deviation of split frequencies <0.01 and the potential scale reduction factors (PSRF) were close to 1.0 for all parameters. We also used Tracer v1.5 (Rambaut & Drummond, 2007) to investigate the convergence of the BI analyses. The ML analyses were implemented in RAxML‐VI‐HPC (Stamatakis, 2006) with the GTR + I + G model. Nodal support values were evaluated from 1,000 nonparametric bootstrap replicates. The best partition scheme (Table S5), as determined by PartitionFinder was used in the phylogenetic analyses.

### 2.5 RAG1 and COI

We aligned RAG1 and COI sequences using MUSCLE. BI and ML analyses were used to infer phylogenetic relationships among Nemacheilidae species using MrBayes 3.1.2 and RAxML‐VI‐HPC, respectively. The optimal substitution model was chosen based on the Akaike information criterion (AIC) in MrModeltest version 2.3. Four independent runs were performed for 50 million generations. The phylogenetic trees were sampled every 1,000th generation and the first 25% were discarded as burn‐ins. The ML analyses were conducted with the GTR + I + G model and 1,000 nonparametric bootstrap replicates.

### 2.6 Divergence time estimation

We estimated node age using the Bayesian phylogenetic software BEAST v1.8.2 (Drummond & Rambaut, 2007). The concatenated sequences containing the 22 tRNA genes, the 1th and 2nd of the 13 PCGs, and the two ribosomal RNA genes were analyzed. An independent GTR model of nucleotide substitution with gamma‐distributed rate variation across sites inferred by MrModeltest (Nylander, 2004) and the uncorrelated lognormal relaxed clock (Drummond, Ho, Phillips, & Rambaut, 2006) were used for this analysis. The initial phylogenetic tree was generated randomly, and the tree prior followed a Yule branching model.

Two fossil calibrations were incorporated in this study. (a) The oldest fossil of *Plesiomyxocyprinus arratiae* similar to *Myxocyprinus asiaticus* was constrained to be from the middle Eocene (38–40 Ma). This calibration point was modelled using a normal distribution with a mean of 39 Ma and standard deviation of 1.0 Ma, providing a 95% confidence interval of 37.76–40.64 Ma (C1). (b) Another fossil calibration of the genus *Cobitis* was 13.8–15.9 Ma (C2). A lognormal prior of 14.9 Ma was taken as the zero offset; the default lognormal standard deviation of 0.04 was used to constrain this node, giving a 95% confidence interval of 13.94–15.9 Ma. Four independent runs were performed using 100 million generations and sampling every 1,000th tree with the same settings. We then log‐combined the two analyses into one dataset using LogCombiner v1.8.0 (Drummond & Rambaut, 2007), as several ESSs of posteriors of an independent run were <200. All runs were checked for sufficient mixing, stable convergence on unimodal posterior, and effective sample sizes for all parameters using TRACER v1.5. Subsequently, after removing 50% of the resulting trees as burn‐in, the resulting trees were summarized in a Maximum Clade Credibility consensus tree with TreeAnnotator v1.8.0 (Drummond & Rambaut, 2007).

## 3 RESULTS

### 3.1 Characteristics of the two *Schistura* mitogenomes

**Figure 1:**
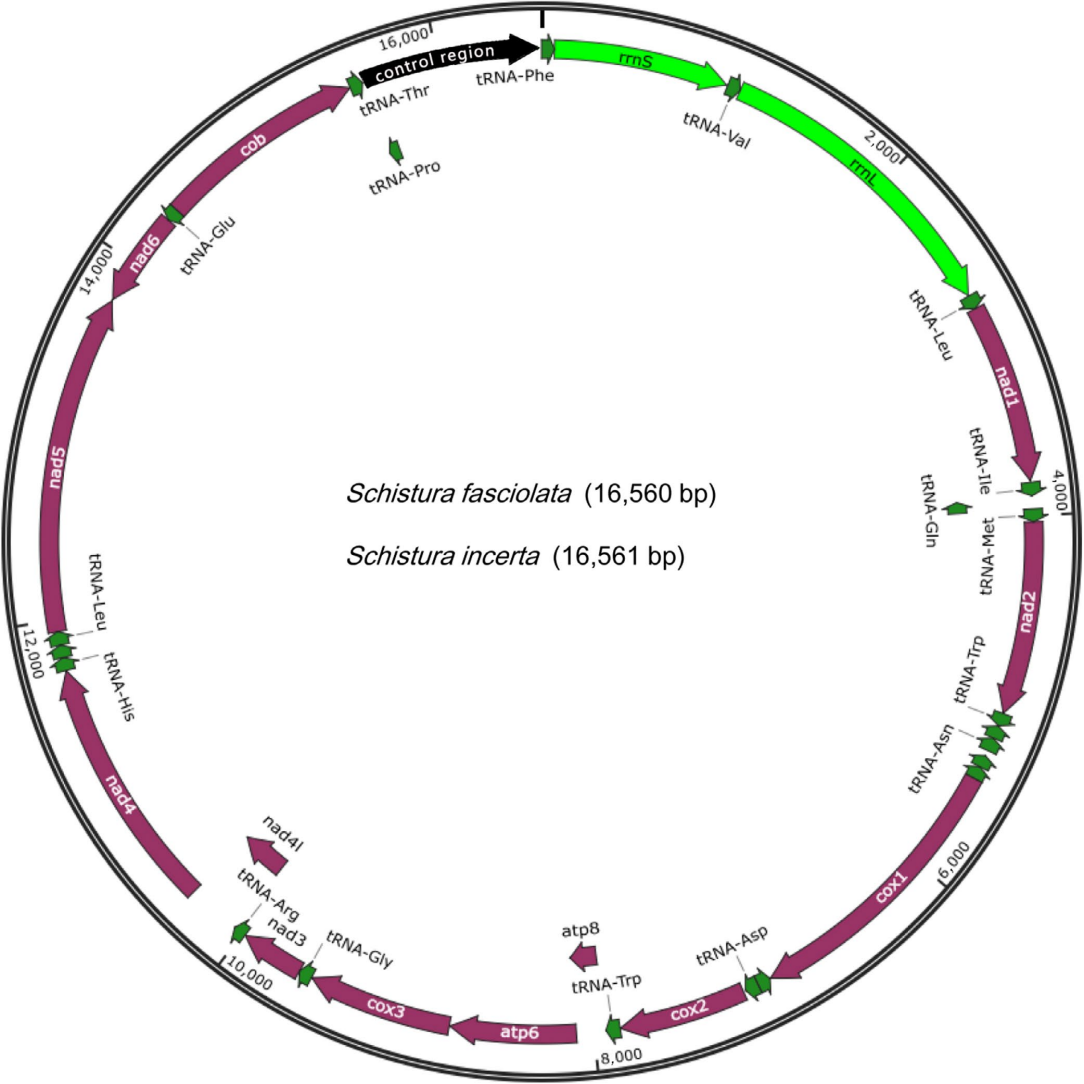
Organization of the two newly sequenced mitochondrial genomes of *Schistura fasciolata* and *Schistura incerta*

The genomes of *S. fasciolata* and *S. incerta* reached 16,560 and 16,561 bp, respectively. Both genomes contained a typical set of 37 mitochondrial genes (13 PCGs, 22 tRNAs and 2 rRNAs) and a putative control region (also known as A+T‐rich region; Figure 1).

### 3.2 Phylogenetic trees based on mitogenomes

**Figure 2:**
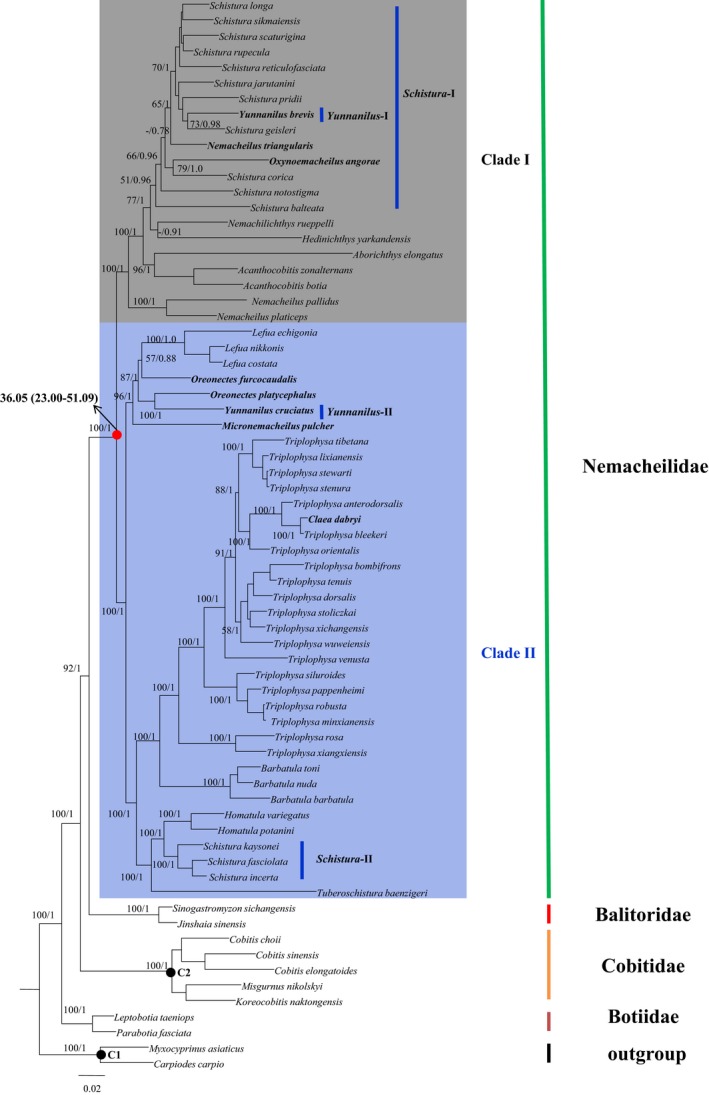
Maximum likelihood tree for 58 Nemacheilidae species based on mtDNA genomes. Numbers near branches indicate bootstrap proportions and Bayesian posterior probabilities from maximum likelihood analysis and Bayesian inferences, respectively. Number in bold with red dot shows the divergence time estimation with 95% confidence interval (CI) between the Nemacheilidae clades. C1 and C2 represent the placement for the two calibrations used in our study

The length of 68 aligned mitochondrial genomes reached 16,011 bp. After discarding the third positions of the 13 PCGs, the remaining bases (12,163 bp) were used for phylogenetic inference and divergence time estimates. BI and ML analyses yielded identical and well‐supported tree topologies in major nodes. They differed only in the support values for certain nodes (Figure 2). The phylogenetic results showed that the family Nemacheilidae has two main clades (I and II; Figure 2), each with high support. Genera *Schistura*, *Petruichthys*, *Mesonoemacheilus*, *Oxynoemacheilus*, *Hedinichthys*, *Nemachilichthys*, *Acanthocobitis*, *Aborichthys*, and *Nemacheilus* grouped into clade I and genera *Triplophysa*, *Claea*, *Barbatula*, *Tuberoschistura*, *Homatula*, *Schistura*, *Micronemacheilus*, *Oreonectes*, and *Lefua* clustered into clade II.

Unexpectedly, several genera did not form the reciprocal monophyly inferred from the ML and BI trees. *Claea dabryi* and *Triplophysa bleekeri* formed a clade that was sister to *T. anterodorsalis*. Representative species from *Petruichthys*, *Oxynoemacheilus*, and *Mesonoemacheilus* were nested within the genus *Schistura* with strong support. *Oreonectes furcocaudalis* first clustered with genus *Lefua* and then was sister to the clade containing *Yunnanilus cruciatus* and* Oreonectes platycephalus* (Figure 2). We found that members of genus *Schistura* were separated between the two major clades. The divergence time between the two clades was estimated to have occurred 36.05 Ma (23.00–51.09 Ma 95% confidence interval [CI]; Figure 2).

### 3.3 Phylogenetic trees based on RAG1 and COI

**Figure 3:**
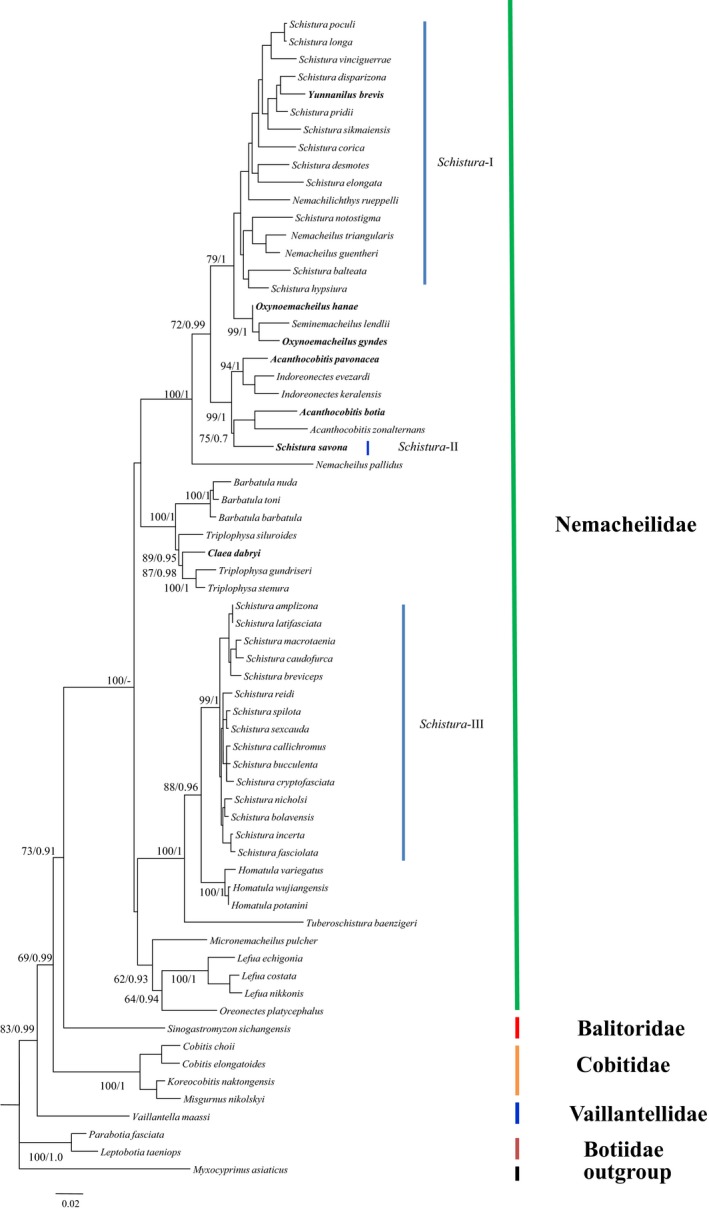
Maximum likelihood tree for 57 Nemacheilidae species based on RAG1 sequences. Numbers near branches indicate bootstrap proportions and Bayesian posterior probabilities from maximum likelihood analysis and Bayesian inferences, respectively

**Figure 4:**
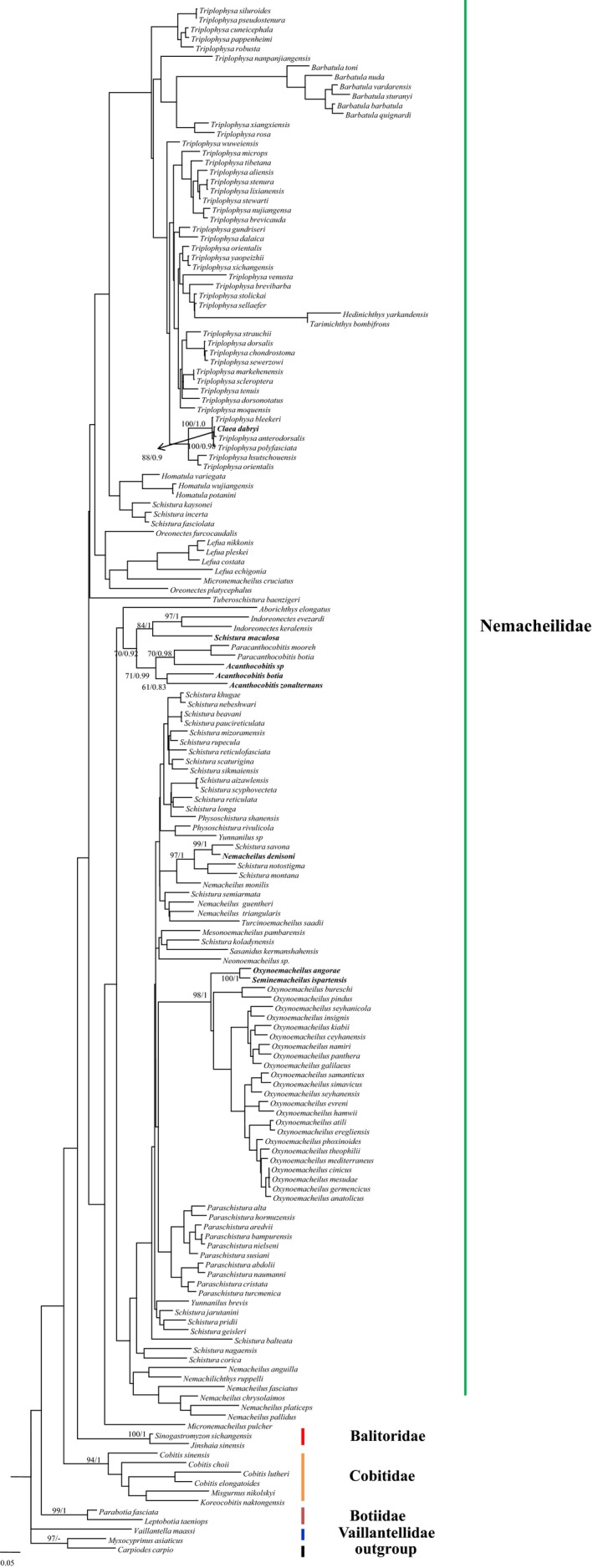
Maximum likelihood tree for 150 Nemacheilidae species based on COI sequences. Numbers near branches indicate bootstrap proportions and Bayesian posterior probabilities from maximum likelihood analysis and Bayesian inferences, respectively (supported values are only shown in some nodes)

The length of aligned RAG1 and COI sequences reached 865 and 626 bp, respectively. Neither BI or ML trees, using RAG1 and COI datasets, showed division of the two clades, which was observed in the mitogenome trees. BI and ML trees, based on RAG1, obtained four clades with the clade including the genera *Micronemacheilus*, *Oreonectes*, and *Lefua* exhibiting relatively low supported values (Figure 3). The relationships among all four clades were not resolved because of comparatively low supported values. This might have been due to the lack of sufficient variable sites. Phylogenetic trees using COI sequences did not show a credible split of clades because relatively low supported values were obtained in most of the nodes (Figure 4).

The RAG1 and COI analyses also showed that several genera were not monophyletic. In RAG1 trees (Figure 3), *Claea dabryi* and *Schistura savona* were nested within the genus *Triplophysa* and the genus *Acanthocobitis*, respectively. *Oxynoemacheilus gyndes* first clustered with *Seminemacheilus lendlii* and then was sister to *Oxynoemacheilus hane*. *Acanthocobitis pavonacea* first clustered with *Indoreonectes* and then was sister to the clade containing *Schistura savona* and two *Acanthocobitis* species (Figure 3). Representative species from *Yunnanilus* and *Nemacheilus* were nested within genus *Schistura*. More importantly, species of genus *Schistura* were found in two highly supported clades. Though the supported values at most nodes in the COI analyses were comparatively low (Figure 4), some genera did not generate reciprocal monophyly. For example, *Claea dabryi* and *Triplophysa anterodorsalis* formed a clade that was sister to other *Triplophysa* species. *Schistura maculosa* clustered with genus *Indoreonectes*. An unidentified *Acanthocobitis* species and genus *Paracanthocobitis* grouped into a clade that was sister to other *Acanthocobitis* species, and *Nemacheilus denisoni* nested within *Schistura*.

### 3.4 Species distributions

**Figure 5:**
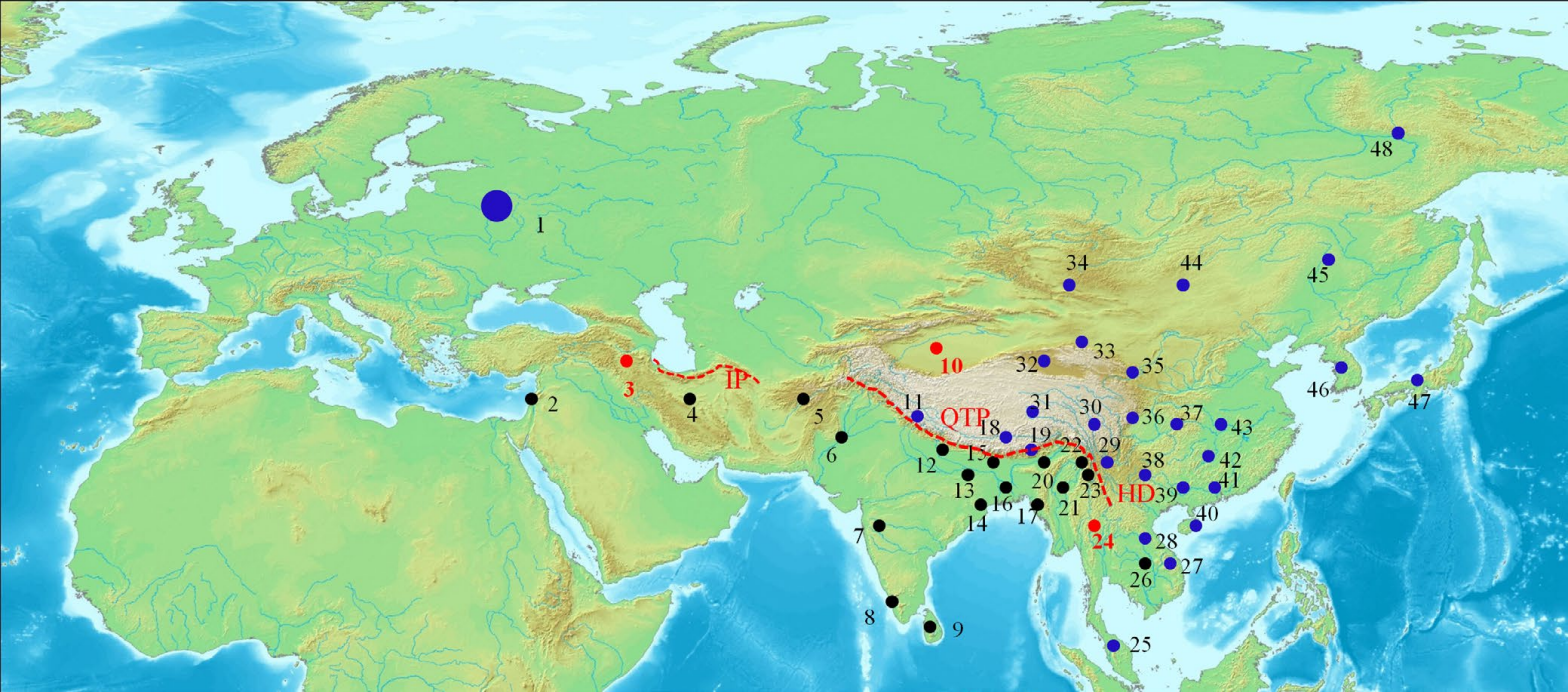
Map showing species distributions of 58 Nemacheilidae species used in the mitogenome analyses. Black and blue dots indicate distribution ranges of species within clade I and species within clade II, respectively. Red dots show distribution ranges of species detected in both clades. Localities of each species are detailed in Table S1. HD, Hengduan Mountains; IP, Iran Plateau; QTP, Tibetan Plateau

Because the phylogenetic topologies utilizing RAG1 and COI datasets were not well resolved, we used species map distributions based on species that had mitogenome sequences (Figure 5). The map suggested a majority of species from clade I were located in South Asia, Southeast Asia, West Asia and the Middle East. Most species in clade II were distributed in East Asia, Europe and the Far East. A few exceptions were found in Iran (Locality 3), Tarim in China (Locality 10), and Thailand (Locality 24). These locations had species that were located in both major clades (Figure 5; Table S1).

## 4 DISCUSSION

### 4.1 Comparisons between molecular phylogenies and morphological classifications

Nemacheilidae contains many morphologically similar species and taxonomic problems exist at species and genus levels (Nalbant & Bianco, 1998; Tang et al., 2006). We studied the phylogenetic positions of several species and genera by analyzing the mitogenome sequences of RAG1 and COI.

Three species, *Yunnanilus brevis*, *Nemacheilus triangularis*, and *Oxynoemacheilus angorae*, were nested within the *Schistura* clade inferred from mitogenome analyses, though support values were relatively low based on ML trees (Figure 2). RAG1 analyses showed that *Y. brevis* and three *Nemacheilus* species (*N. triangularis*, *N. guentheri*, and *N. rueppelli*) were nested within the *Schistura* species complex. *Yunnanilus brevis*, within the *Schistura* clade, was also examined by Sgouros (2016). The present findings also challenged the monophyly of *Schistura*. Although the abovementioned species currently are placed in different genera, they were once considered to be in *Nemacheilus*, as well as the *Schistura* species (http://www.fishbase.com). This suggests that earlier studies on *Schistura*, *Nemacheilus*, *Oxynoemacheilus*, or *Yunnanilus* found insufficient morphological evidence to support the recognition of these genera as a natural grouping. Some genera within the Nemacheilidae, for example *Schitura*, have many species that are difficult to delimit due to the absence of stable morphological characters (Bǎnǎrescu & Nalbant, 1995). Future morphological studies should consider using our phylogenetic data for species differentiation.

Three *Nemacheilus* species in this study segregated into two different subclades within clade I (Figure 2). A similar pattern was seen in the RAG1 analyses. *N. triangularis* and *N. guentheri* are a valid name in the FishBase database (http://www.fishbase.com), but researchers have used *Mesonoemacheilus triangularis* and *M. guentheri* in their studies (Anoop, Dahanukar, Ali, & Raghavan, 2017; Raghavan, Prasad, Ali, & Pereira, 2008; Slechtova, Bohlen, & Tan, 2007). Consequently, we propose that *N. triangularis* and *N. guentheri* belong in *Mesonoemacheilus*.

*Claea dabryi* nested with *Triplophysa* species based on three molecular datasets. This was consistent with the work of Liu et al. (2012) and Li, Yang, Si, Zhang, and Song (2016). The position of *C. dabryi* indicated that this is a young species (1.37 Ma; not shown in Figure 2) derived from *Triplophysa* species, and challenged the monophyly of genus/subgenus *Triplophysa* supported by He, Chen, and Chen (2006) and Wang et al. (2016).

Our study shows that *Oreonectes* does not form monophyletic clade. *Oreonectes furcocaudalis* formed a clade with *Lefua*, whereas *Oreonectes platycephalus* generated another clade with *Yunnanilus cruciatus*. Previous phylogenetic studies on Nemacheilidae species did not recognize this phylogenetic pattern because they included only a limited number of species from *Lefua* and *Oreonectes* and did not contain *Yunnanilus* species (Liu et al., 2012, 2010; Tang et al., 2006). The diagnostic morphological difference between *Oreonectes* and *Lefua* is that *Lefua* has a vertical and brown line from mouth to the base of tail fin, whereas *Oreonectes* lack this line (Zhu, 1989). *Oreonectes* and *Yunnanilus* differ in their head shape and the length of fistuliform prominence of the anterior nares (Zhu, 1989). The morphological differences and nonmonophyletic clade indicate that *Oreonectes* may not have an exclusive origin. Further study of this classification is needed by examining more *Oreonectes* species and additional morphological and molecular characters. RAG1 and COI analyses did not provide relevant evidence due to the absence of *Oreonectes furcocaudalis* and low supported values.

Evidence for paraphyly in *Oxynoemacheilus* and *Acanthocobitis* was inferred from RAG1 and/or COI analyses (Figures 3 and 4). Trees using RAG1 and COI with individual *Oxynoemacheilus* species shared a clade with *Seminemacheilus* species. Stoumboudi, Kottelat, and Barbieri (2006) and Prokofiev (2009) placed most Nemacheilidae from Eastern Europe and the Middle East into the genus *Oxynoemacheilus*, including the genus *Seminemacheilus*. This suggested that *Oxynoemacheilus* or *Seminemacheilus* lacked sufficient morphological evidence to separate the two genera. A similar pattern occurred in *Acanthocobitis*, *Indoreonectes*, and *Paracanthocobitis*. They were placed in the genus *Nemacheilus* (http://www.fishbase.com). This phylogenetic pattern might be due to the uncertain division between these genera.

*Schistura* species fell into two major clades based on mitogenomic data. In addition to the phylogenetic position of the *Schistura* clade within clade I, polyphyly of *Schistura* was verified based on a larger dataset. *Schistura* species formed two independent clades in the RAG1 topologies as well. In addition, many species from other genera nested within the *Schistura* species complex and individual *Schistura* species (e.g., *S. savona* and *S. maculosa*) nesting within other genera are indicative of the polyphyly of *Schistura*. The polyphyly of *Schistura* has been noted in other studies (Liu et al., 2012, 2010; Nalbant & Bianco, 1998; Sgouros, Page, Orlofske, & Jadin, 2019; Tang et al., 2006). For example, Tang et al. (2006) reported two paraphyletic clades based on two mtDNA gene fragments. Liu et al. (2012) found two paraphyletic clades based on two mitochondrial gene sequences and four nuclear gene segments. Siva et al. (2017) detected two paraphyletic clades based on the entire mitochondrial genome. *Schistura* has 200 known species and occupies a vast geographical range. It has considerable morphological diversity which increases the difficulty of accurate species identification. We believe that *Schistura* should be divided into at least two genera. Additional studies are needed to identify rigorous synapomorphies for the two hypothetic genera based on molecular characters. A similar pattern was found in *Yunnanilus* (Figure 2). *Yunnanilus brevis* was regarded as a synonym of *Eonemachilus brevis* and *Nemacheilus brevis*, and *Yunnanilus cruciatus* was treated as a synonym of *Nemacheilus cruciatus* and *Micronemacheilus cruciatus*. This suggests that the genus‐level classification of these two species is problematic. The phylogenetic relationships seen in the current study suggests that the two species belong in different genera. Additional *Yunnanilus* species are needed to resolve the complex relationships within this genus.

The phylogenetic relationships of many species and genera may not be consistent with the traditional morphological subdivision of the Nemacheilidae. Many taxonomic problems exist in this family. More work on morphological traits and phylogenetic inferences should be done to understand the complicated relationships within the Nemacheilidae. The present study provides useful information for future researches.

### 4.2 Diversification related to the uplift of the Tibetan Plateau

The Nemacheilidae consist of two highly supported clades (I and II). Members of clade I primarily reside in India and central Asia, and members of clade II are mainly distributed in East Asia, Tibetan Plateau, and Europe (Figure 3). Tibetan Plateau, Hengduan Mountains, and Iran Plateau seem to form a boundary between the two clades. Molecular dating indicates that the Nemacheilidae diversified at 36.05 Ma (23.00–51.09 Ma), suggesting that the two clades separated sometime during the Late Eocene and the Oligocene. This phase broadly agrees with the first upliftment of the Tibetan Plateau (~40 Ma), accompanied by the rise of high mountains, the shaping of many river networks, and a greatly modified physiognomy of the plateau and adjacent regions (Chung et al., 1998; Rowley & Currie, 2006). These new mountains and river networks became geographic barriers and diversification reservoirs for Nemacheilidae species, resulting in differentiation. Similar effects have been reported for Asiatic salamanders (Zhang et al., 2006). Thus, the geological history of the Tibetan Plateau appears to be responsible for driving the diversification of Nemacheilidae taxa.

## CONFLICT OF INTEREST

All authors declare that they have no competing interests.

## AUTHORS' CONTRIBUTIONS

WC and XL designed the research and WC wrote the manuscript. JY and YL performed sampling. All authors read and approved the final manuscript.

## Data Availability

DNA sequences have been deposited in GenBank under Accession numbers KY404236, MK361215, and MN062061–MN062062. Details regarding individual samples are available in Table S1.
