# Supplementary material for: Exploring taxonomic diversity and biogeography of the family Nemacheilinae (Cypriniformes)
Source: Ecol Evol. 2019 Aug 28;9(18):10343–53. doi: 10.1002/ece3.5553 (PMC6787813; doi:10.1002/ece3.5553)
Supplement: Supplementary file 4 [file ECE3-9-10343-s004.docx]

| Gene regions | Symmetrical tree | | | Asymmetrical tree | | |
| --- | --- | --- | --- | --- | --- | --- |
|  | *Iss* | *Iss.c* | *P* | | *Iss.c* | *P* |
| all positions of 13 PCGs | 0.247 | 0.818 | < 0.0001 | | 0.572 | < 0.0001 |
| (1st + 2nd) positions of 13 PCGs | 0.084 | 0.815 | < 0.0001 | | 0.571 | < 0.0001 |
| 1st positions of 13 PCGs | 0.129 | 0.809 | < 0.0001 | | 0.555 | < 0.0001 |
| 2nd positions of 13 PCGs | 0.040 | 0.809 | < 0.0001 | | 0.555 | < 0.0001 |
| **3rd positions of 13 PCGs** | **0.602** | **0.809** | **< 0.0001** | | **0.555** | **< 0.0001** |
| 12s rRNA | 0.217 | 0.747 | < 0.0001 | | 0.442 | < 0.0001 |
| 16s rRNA | 0.288 | 0.783 | <0.0001 | | 0.509 | < 0.0001 |
| 22 tRNAs | 0.376 | 0.784 | < 0.0001 | | 0.510 | = 0.0003 |
