# Supplementary material for: Exploring taxonomic diversity and biogeography of the family Nemacheilinae (Cypriniformes)
Source: Ecol Evol. 2019 Aug 28;9(18):10343–53. doi: 10.1002/ece3.5553 (PMC6787813; doi:10.1002/ece3.5553)
Supplement: Supplementary file 5 [file ECE3-9-10343-s005.docx]

| Scheme Number | Composition | Best model |
| --- | --- | --- |
| 1 | 1st codon positions of 13 PCGs | GTR+I+G |
| 2 | 2nd codon positions of 13 PCGs | GTR+I+G |
| 3 | 12s rRNA & 16s rRNA | GTR+I+G |
| 4 | 22 tRNAs | TVM+I+G |
